# Explainable Superpixel-Guided Graph Vision Transformer for Hyperspectral Image Analysis

**DOI:** 10.3390/s26175513

**Published:** 2026-08-31

**Authors:** Jieli Chen, Kah Phooi Seng, Chee Shen Lim, Li-Minn Ang, Jeremy Smith

**Affiliations:** 1 School of Advanced Technology, Xian Jiaotong-Liverpool University, Suzhou 215123, China; 2 Department of Electrical Engineering & Electronics, University of Liverpool, Liverpool L69 3BX, UK; j.s.smith@liverpool.ac.uk; 3 Faculty of Innovation and Technology, Taylor’s University, Selangor 47500, Malaysia; 4 School of Science, Technology and Engineering, University of Sunshine Coast, Petrie, QLD 4502, Australia

**Keywords:** superpixel vision transformer, explainable artificial intelligence, hyperspectral image classification

## Abstract

Hyperspectral imaging provides rich spectral–spatial information for fine-grained material discrimination, but effective and interpretable modeling remains challenging because land-cover regions often have irregular spatial structures and class-specific spectral responses. Conventional methods typically rely on fixed grid patches or local neighborhoods, which may not align with natural object boundaries, whereas pure superpixel or graph models may lose fine pixel-level details. This paper proposes an explainable superpixel-guided graph vision transformer (ESG-ViT) for hyperspectral image classification and analysis. The proposed framework contains two complementary branches: a graph superpixel vision transformer (GS-ViT) that represents hyperspectral scenes as adaptive superpixel tokens and injects graph topology into self-attention, and a windowed pixel vision transformer (WP-ViT) that preserves dense local spectral–spatial details through efficient local attention. The two representations are adaptively fused for pixel-wise classification. To support interpretability, the model further derives class-wise superpixel relevance maps and spectral channel importance from gradient responses, revealing both the spatial regions and wavelength channels that contribute to each category. Experiments on multiple benchmark hyperspectral datasets demonstrate that the proposed method improves classification accuracy while producing clearer, more human-aligned explanations. Visualization results show that the model highlights meaningful class-related superpixel regions and assigns distinct spectral-channel importance patterns to different classes. These results indicate that the proposed framework provides an accurate and explainable alternative to conventional patch-based transformers for hyperspectral image analysis.

## 1. Introduction

Hyperspectral imaging records the reflectance of a scene across a large number of contiguous spectral bands. Compared with conventional imagery, hyperspectral images (HSIs) provide detailed spectral responses that support the identification of materials with similar visual appearances [1]. HSI classification has therefore been widely studied in applications such as agricultural monitoring, environmental analysis, mineral exploration, and urban land-cover mapping [2,3]. However, this task remains challenging because HSIs have high spectral dimensionality, limited labeled samples, complex spatial structures, and spectral variability within the same category [1].

Early HSI classification methods mainly relied on spectral features and traditional classifiers, such as support vector machines and sparse representation models [4]. Subsequent convolutional neural network (CNN) methods improved classification by jointly learning spectral and spatial information from local image neighborhoods [5,6]. Although CNNs are effective for local feature extraction, their receptive fields are usually defined by fixed square kernels. Such regular neighborhoods do not always match the irregular boundaries of land-cover regions. Graph convolutional networks (GCNs) address this limitation by representing an HSI as a graph and propagating information among adjacent regions. In particular, superpixel-based graphs reduce computational cost by grouping neighboring pixels into spatially coherent regions [7,8]. However, region aggregation may remove useful pixel-level details, especially near boundaries or within small objects.

Vision transformers (ViTs) provide another approach to spatial-context modeling by using self-attention to capture dependencies among visual tokens [9]. Standard ViTs usually divide an image into fixed grid patches. While this representation is convenient, regular patches may cross natural region boundaries and mix pixels belonging to different materials. Applying global attention directly to all HSI pixels is also computationally expensive because the number of tokens increases rapidly with image size. These limitations suggest that a single token representation is insufficient: region-level tokens are useful for structural reasoning, while dense pixel-level tokens remain necessary for preserving local spectral–spatial information.

Explainability is also important for HSI analysis. In practical applications, a classification map alone does not reveal why a model assigns a category to a region. It is useful to determine whether the prediction is supported by relevant spatial areas and whether different categories depend on different spectral channels. Existing explainability methods often focus on pixel-level saliency or general attention visualization [10,11]. However, pixel-level maps can be fragmented, and attention weights alone do not directly quantify how the input spectrum affects a class response. An explanation framework for HSI classification should therefore connect spatial regions, graph interactions, and spectral-channel contributions.

**Figure 1:**
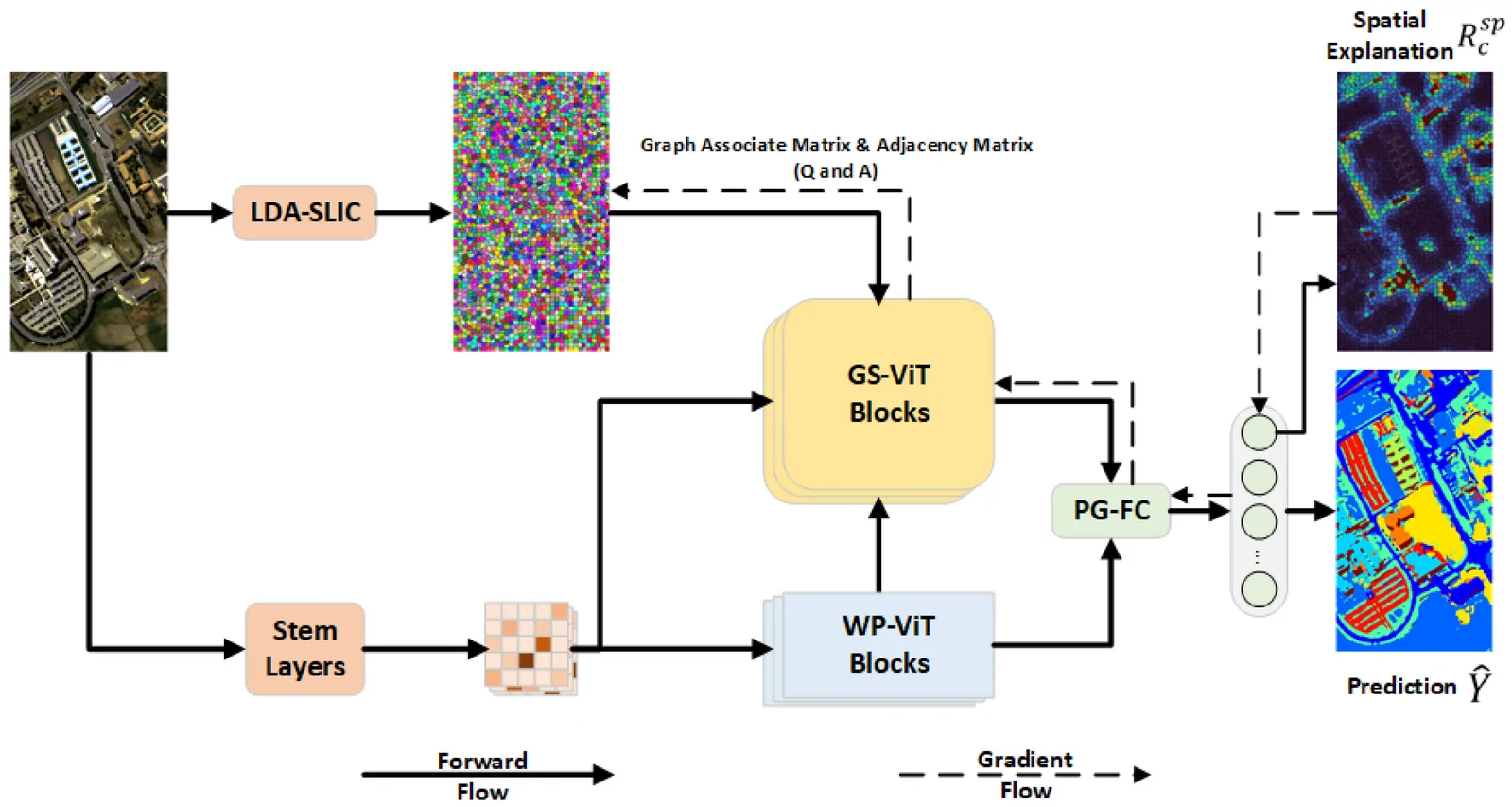
Proposed graph superpixel vision transformer with explainable analysis for hyperspectral image.

To address these issues, we propose an Explainable Superpixel-Guided Graph Vision Transformer (ESG-ViT) for hyperspectral image classification. As shown in Figure 1, ESG-ViT uses a dual-branch architecture to model complementary spectral–spatial information. The first branch, named Graph Superpixel Vision Transformer (GS-ViT), represents the HSI as a graph of adaptive superpixel tokens. The superpixel construction follows an existing segmentation strategy [7], while the main design of GS-ViT lies in embedding the resulting graph structure into transformer reasoning. Instead of using graph propagation as an independent post-processing step, GS-ViT injects the normalized superpixel adjacency matrix into the self-attention logits. The graph prior encourages stronger interaction among adjacent and similar regions while retaining the adaptive token interaction of a transformer. The second branch, termed the Windowed Pixel Vision Transformer (WP-ViT), operates on dense pixel-level tokens. WP-ViT restricts self-attention to local windows and uses shifted windows across successive layers. This design reduces the cost of dense pixel modeling while allowing information exchange between neighboring windows. GS-ViT and WP-ViT play different roles: GS-ViT captures region-level context over irregular structures, while WP-ViT preserves local boundaries and spectral–spatial variations that may be weakened by superpixel aggregation. Their outputs are aligned on the original image grid and combined through adaptive pixel–graph transformer fusion.

ESG-ViT further provides explanations at both spatial and spectral levels. For spatial interpretation, class-specific input gradients are aggregated within superpixel regions to produce coherent region relevance maps. These maps show which regions contribute most strongly to each category. For spectral interpretation, gradient responses are accumulated across spatial positions to estimate the importance of each spectral channel for each class. The resulting class-channel heatmaps show that different categories rely on different spectral responses.

The main contributions of this work are summarized as follows:

- First, ESG-ViT introduces a dual-branch transformer architecture that combines graph–superpixel modeling with pixel-level complementary for HSI classification.
- Second, GS-ViT integrates the superpixel graph directly into self-attention, allowing graph topology to guide region-level feature interaction jointly.
- Third, WP-ViT preserves dense local information with efficient windowed attention and complements the region-level graph representation.
- Finally, the proposed explainability analysis derives class-specific superpixel relevance maps and spectral-channel importance heatmaps, providing spatial and spectral evidence for model predictions.

## 2. Related Work

### 2.1. Hyperspectral Image Classification

Hyperspectral image classification requires the joint modeling of spectral responses and spatial context. CNN-based methods improve classification accuracy by exploiting local neighborhoods, but their receptive fields are usually defined by regular kernels. These kernels do not naturally describe land-cover regions with irregular boundaries [5,6].

Graph neural networks provide an alternative representation by propagating information between spatially adjacent pixels or regions [12,13]. Superpixel-based graphs reduce computational cost by grouping neighboring pixels into coherent regions and treating each region as a graph node. CEGCN combines pixel-level CNN features with superpixel-level graph features [7]. MSG-SGAT constructs multistage superpixel graphs and applies sparse graph attention [14]. CEGAT further combines convolutional features with an enhanced graph-attention branch under limited-label conditions [8]. These studies demonstrate the value of region-level graph representations. However, superpixel aggregation may remove useful local variations near boundaries and within small objects. In addition, graph propagation and attention modeling are often implemented as separate stages.

More recently, hybrid neural networks that combine different structural architectures (e.g., CNNs, GCNs, and Vision Transformers) have gained considerable popularity in HSI analysis. For instance, Zhong et al. [15] proposed a dual-branch subpixel-guided network to handle fine spatial details alongside spectral signatures. Similarly, a global multiscale hybrid network was introduced by Wang et al. [16] to integrate multi-scale local features with global contextual dependencies. To capture structural relationships among irregular land cover features, GCGV [17] combines graph attention, CNNs, and transformers into a unified dual-branch network. Furthermore, hybrid architectures coupling Vision Transformers with Graph Convolutional Networks (GCNs) [18] have successfully bridged grid-like pixel domains and graph-like non-Euclidean structures for HSI classification. Unlike these hybrid networks that typically merge features from parallel branches at the decision level, ESG-ViT uniquely integrates grid-like and graph-like structures by embedding the irregular superpixel graph topology directly into the Transformer’s self-attention matrix. This allows the model to capture region-level context over irregular structures while retaining dense pixel-level local details and providing inherent multi-level explainability.

ESG-ViT addresses these limitations through two complementary branches. GS-ViT inserts the superpixel graph topology directly into transformer attention, while WP-ViT retains dense pixel-level information through local windowed attention. The proposed method therefore uses superpixels as an adaptive structural representation without relying exclusively on region-level features.

### 2.2. Vision Transformers for Hyperspectral Images

Transformers have attracted increasing interest in hyperspectral image classification because self-attention can model dependencies beyond local convolutional neighborhoods [9,19]. SpectralFormer treats spectral signatures as sequences and introduces group-wise spectral embeddings with cross-layer adaptive fusion [20]. SSFTT combines convolutional feature extraction with spectral–spatial tokenization and transformer encoding [21].

Standard vision transformers usually generate tokens from fixed grid patches. Such patches may cross natural region boundaries and mix pixels belonging to different materials. However, applying global attention directly to dense pixel tokens also increases computational cost. Recent methods have introduced multiscale, sparse, or hierarchical attention mechanisms to address these issues [22,23]. Superpixel-based tokenization provides another direction by replacing fixed grid partitions with adaptive regions [24,25]. ESG-ViT extends this idea for hyperspectral analysis by representing superpixels into a graph structure, injecting adjacency information into self-attention, and retaining a parallel windowed pixel branch.

In this context, modern Vision Transformers specifically tailored for HSI have redefined spectral–spatial feature learning. For instance, SpectralFormer [20] leverages group-wise spectral embeddings and cross-layer adaptive fusion, while SSFTT [21] combines 3D/2D CNN stems with a Gaussian-weighted tokenization transformer. ESG-ViT extends these transformer architectures by representing superpixels as a graph structure and directly embedding the adjacency prior into the self-attention mechanism. Furthermore, as highlighted in the meta-analysis by Kasetty and Krishnan [26], transform-aware deep learning architectures integrated with explainable AI (XAI) mechanisms represent a critical paradigm shift in remote sensing, enabling models to extract complex spatial–spectral representations while offering transparent decision boundaries.

### 2.3. Explainable Hyperspectral Image Analysis

Explainability remains less developed than classification accuracy in hyperspectral image analysis. Existing approaches commonly use gradient responses or SHAP-based methods to estimate the contribution of input pixels and spectral channels [27,28,29,30]. In land use and land cover (LULC) classification, SHAP-based interpretable frameworks have been successfully applied by Temenos et al. [31] to quantify feature contributions and land-cover boundary influences. However, feature-level SHAP values often entail heavy sampling overheads when scaled to high-dimensional hyperspectral cubes. To make XAI feasible for resource-constrained edge computing environments, De Lucia et al. [32] demonstrated that explainability mechanisms must be co-designed with computationally efficient model backbones. Band-level explanations are useful because different materials may depend on different wavelength ranges. However, channel importance alone does not explain where the model identifies class-related evidence in the scene. Attention visualization provides another form of interpretation, but raw attention weights do not fully describe the contribution of input features to a prediction. Attention rollout and attention flow have been proposed to trace interactions through stacked transformer layers [33]. ESG-ViT combines graph-aware attention analysis with gradient-based explanations. Class-specific gradients are aggregated within superpixel regions to produce spatially coherent relevance maps and accumulated across spatial positions to estimate spectral-channel importance. This provides complementary spatial and spectral evidence for each class prediction.

## 3. Methodology

### 3.1. Overall Architecture

Hyperspectral image analysis requires a representation that can simultaneously preserve fine spectral–spatial details at the pixel level and exploit the structural consistency of larger homogeneous regions. Pixel-wise deep models are effective for modeling local spectral variations, but they usually process the image on a regular Euclidean grid and may require expensive dense interactions to capture broad spatial context. In contrast, superpixel- and graph-based methods provide a compact representation of spatially coherent regions and are well suited to irregular land-cover structures [7,8]. However, if the image is represented only by superpixel nodes, fine boundaries and within-region spectral variations may be weakened by region averaging. Motivated by these complementary strengths and limitations, the proposed framework adopts a dual-branch transformer design. One branch performs graph-aware reasoning over superpixel tokens, while the other performs local self-attention over dense pixel tokens. The resulting architecture aims to combine region-level semantic consistency, pixel-level detail preservation, and built-in explainability.

Let the input hyperspectral image (HIS) be denoted by $X\in R^{H\times W\times B}$, where H and W are the spatial dimensions and B denotes the number of spectral bands. The goal is to estimate a pixel-wise class probability map $\hat{Y}\in R^{N\times C}$, where N=H×W denotes the number of pixels and C is the number of land-cover classes. Before transformer encoding, the original spectral cube is standardized and passed through a lightweight spectral transformation stem:

(1) $$F=\phi_{stem}\left(X\right),\;\;F\in R^{H\times W\times D}$$

where $\phi_{stem}\left(\cdot \right)$ denotes the learnable spectral projection and D is the latent feature dimension. In the implementation, D is set to 128.

The first main branch is the Graph Superpixel Vision Transformer, denoted as GS-ViT. A superpixel partition is used to convert the image into a set of irregular but spatially coherent regions. Let $Q\in {\left\{0,1\right\}}^{N\times M}$ denote the pixel-to-superpixel assignment matrix, where M is the number of superpixels. The graph–superpixel branch uses Q to aggregate dense latent features into superpixel tokens and uses the superpixel adjacency matrix $A\in R^{M\times M}$ to describe spatial and spectral neighborhood relationships among regions, where the superpixel construction follows an existing LDA-SLIC style strategy [7]. In GS-ViT, superpixel tokens interact through self-attention whose attention scores are biased by the graph structure, allowing the model to retain the flexibility of transformer attention while respecting the spatial organization encoded by A.

The second main branch is the Windowed Pixel Vision Transformer, denoted as WP-ViT. This branch operates directly on the dense latent feature map F and regards each pixel location as a fine-grained token. Applying global self-attention to all H×W pixels is usually impractical for hyperspectral scenes because the number of tokens can be very large. Therefore, inspired by [19], WP-ViT adopts local window self-attention, together with shifted windows across successive layers, to capture local spectral–spatial dependencies efficiently. This branch is responsible for preserving details that may be lost during superpixel aggregation, including class boundaries, small objects, and within-superpixel spectral variation.

The two branches produce complementary dense representations. The graph–superpixel branch outputs a pixel-restored graph contextual feature $Z_{g}\in R^{N\times D}$, where each pixel receives the graph-enhanced representation associated with its superpixel; the windowed pixel branch outputs a dense pixel feature $Z_{p}\in R^{N\times D}$, which retains local spatial detail and spectral discriminability. The prediction module then adaptively combines these two representations and generates the final class probability map:

(2) $$\hat{Y}=softmax\left(f_{cls}\left(f_{fuse}\left(Z_{p},Z_{g}\right)\right)\right)$$

where $f_{fuse}\left(\cdot \right)$ denotes the adaptive pixel–graph transformer fusion operation, and $f_{cls}(\cdot )$ denotes the final pixel-wise classifier.

Explainability is not attached as a post hoc visualization independent of the model design. Instead, it is supported by the same hierarchical representations used for classification. The graph branch naturally provides region-level evidence because each superpixel token corresponds to a spatially coherent image region. Attention flow [33] among graph–superpixel tokens can therefore be projected back to the image plane, producing interpretable maps that indicate which regions contribute most strongly to the prediction. In addition, gradients with respect to the input spectral bands provide channel-level importance scores, allowing the model to identify which wavelengths are most influential for each class. Consequently, the proposed framework produces explanations at three aligned levels: graph–superpixel attention for region-level reasoning, pixel-level relevance for local spatial evidence, and spectral-band relevance for material-sensitive interpretation.

### 3.2. Graph Superpixel Vision Transformer (GS-ViT)

GS-ViT is designed to model hyperspectral images at the superpixel level. A pixel-level representation preserves local details, but it does not explicitly encode the irregular structure of land-cover regions. Superpixels reduce this redundancy by grouping neighboring pixels with similar spectral–spatial properties, whereas a graph structure can then describe the adjacency and similarity between these regions [7,8,13]. Different from a standard graph convolution, which mainly propagates features along predefined edges, GS-ViT uses the graph as a bias inside self-attention. This keeps the flexible token interaction of a transformer while adding a spatial prior from the superpixel graph.

Given the original input HIS $X\in R^{H\times W\times B}$, we perform dimensionality reduction via LDA and apply the SLIC algorithm to partition the image into M superpixels. Let $Q\in {\left\{0,1\right\}}^{N\times M}$ (where N=H×W) be the binary pixel-to-superpixel assignment matrix. The assignment matrix Q is then used to aggregate the latent feature map $X_{0}\in R^{H\times D_{stem}}$ output by the spectral stem. The column-normalized assignment matrix $\bar{Q}\in R^{N\times M}$ is defined element-wise as:

(3) $${\bar{Q}}_{im}=\frac{Q_{im}}{\sum_{k=1}^{N}Q_{km}}=\frac{Q_{im}}{\left|R_{m}\right|}$$

where ${\bar{Q}}_{im}\in \left\{0,1\right\}$ indicates whether pixel i belongs to superpixel region $R_{m}$, and $\left|R_{m}\right|$ represents the total pixel count in superpixel m.

The initial superpixel token is not built from mean pooling alone. For superpixel region $R_{m}$, GS-ViT combines average and maximum responses:

(4) $$t_{m}^{0}=\left[Avg\left(\left\{u_{i}|i\in R_{m}\right\}\right),\;Max\left(\left\{u_{i}|i\in R_{m}\right\}\right)\right]W_{e}+p_{m}$$

where $u_{i}\in R^{1\times D_{stem}}$ denotes the projected stem feature at superpixel i, [⋅,⋅] denotes feature concatenation along the channel dimension, $W_{e}\in R^{2D_{stem}\times D}$ is the projection weight matrix used to embed the pooled superpixel features into the transformer’s hidden dimension D, and $p_{m}\in R^{1\times D}$ represents the learnable position encoding for the $m^{th}$ superpixel token. Both $W_{e}$ and $p_{m}$ are parameters initialized using Xavier normal distribution (with zero mean and standard deviation of $\sqrt{2/(2D_{stem}+D)}$ and are dynamically updated via backpropagation during end-to-end training of the network.

**Figure 2:**
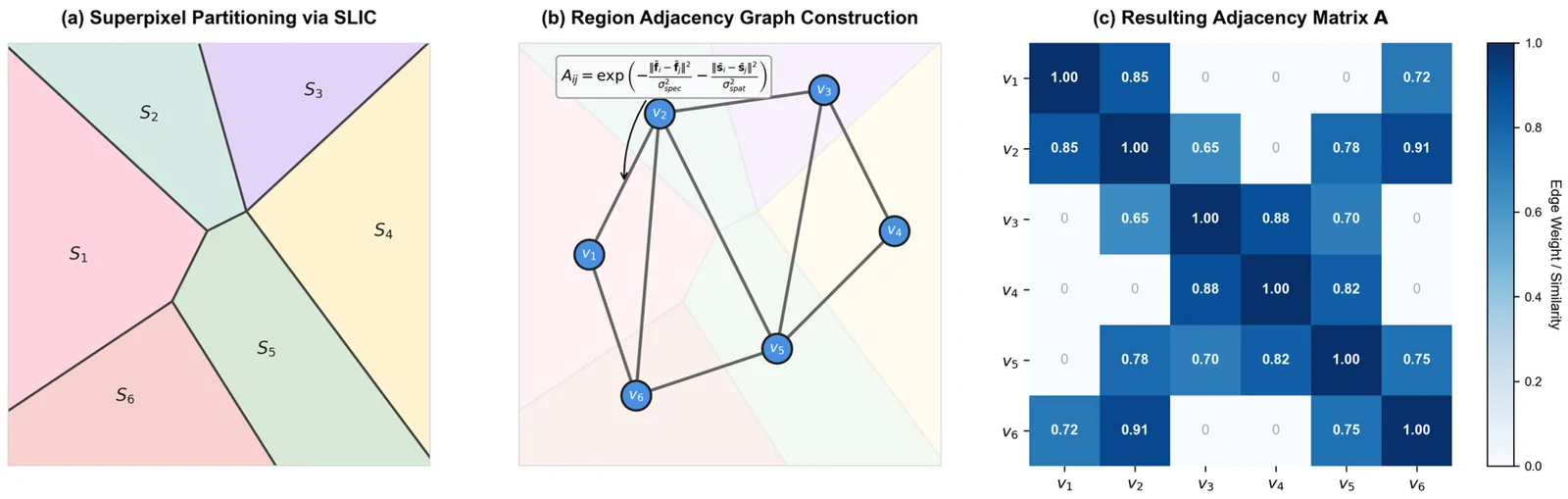
Schematic diagram of the superpixel adjacency matrix construction: (**a**) superpixel partitioning via SLIC, (**b**) region adjacency graph with spatial–spectral RBF kernel weights, and (**c**) the resulting weighted adjacency matrix A.

The graph is defined by the weighted adjacency matrix $A\in R^{M\times M}$. The element $A_{ij}$ is nonzero when superpixel i and superpixel j are spatially adjacent. Its value reflects their spatial–spectral similarity, computed using a Gaussian radial basis function (RBF) kernel:

(5) $$A_{ij}=\exp\left(-\frac{{\left|{\bar{f}}_{i}-{\bar{f}}_{j}\right|}^{2}}{\sigma_{spec}^{2}}-\frac{{\left|{\bar{s}}_{i}-{\bar{s}}_{j}\right|}^{2}}{\sigma_{spat}^{2}}\right)$$

where ${\bar{f}}_{i}\in R^{B}$ is the mean spectral feature vector of superpixel i, ${\bar{s}}_{i}\in R^{2}$ represents its spatial center coordinates, and $\sigma_{spec}^{2}$ and $\sigma_{spat}^{2}$ are scaling parameters. The detailed process of the superpixel partitioning, region adjacency graph (RAG) construction, and the resulting weighted adjacency matrix is visually illustrated in Figure 2. Before A is used in attention, node self-loops are added and the matrix is normalized:

(6) $$A_{g}=\frac{A+I}{\max\left(A+I\right)}$$

where $I\in R^{M\times M}$ denotes the identity matrix representing node self-loops, and max(⋅) denotes the maximum element-wise value of A+I, ensuring that entry values in $A_{g}$ are bounded in $\left[0,1\right]$. Thus, $A_{g}$ provides a bounded graph prior for the transformer layers. For the $h^{th}$ attention head in layer l, GS-ViT computes graph-biased attention as:

(7) $$P_{h}^{l}=softmax\left(\frac{Q_{h}^{l}{\left(K_{h}^{l}\right)}^{T}}{\sqrt{d_{h}}}+\beta_{h}A_{g}+M_{valid}\right)$$

where $Q_{h}^{l}$ and $K_{h}^{l}$ denote the query and key projections of the superpixel tokens, $d_{h}$ is the head dimension, $\beta_{h}$ is a learnable graph-bias coefficient, and $M_{valid}$ is the attention mask matrix enforcing validity constraints. The first term measures feature similarity between tokens. The second term increases the attention score between graph-connected superpixels. Therefore, the graph does not replace self-attention; it guides the attention distribution.

To reduce the information loss caused by superpixel pooling, GS-ViT also merges pixel-level evidence into the graph token stream. Let $Z_{p}$ be the pixel representation pooled by the same superpixel assignment. The fusing update can be represented as:

(8) $${\tilde{T}}^{l}=T^{l}+\alpha \sigma \left(W_{g}\left[T^{l},{\bar{Q}}^{T}Z_{p}\right]\right)⊙{\bar{Q}}^{T}Z_{p}$$

where α denotes a learnable scale, $\sigma \left(\cdot \right)$ is the sigmoid function, ⊙ denotes element-wise multiplication, and ${\bar{Q}}^{T}Z_{p}$ denotes the aligned representation from pixel- to superpixel-level. This gate lets each superpixel token selectively use pixel-level information before graph attention. The graph-biased attention output is then followed by the transformer residual update:

(9) $$T^{l+1}={\tilde{T}}^{l}+Proj\left(P_{h}^{l}V_{h}^{l}\right)+MLP\left(LN\left({\tilde{T}}^{l}+Proj\left(P_{h}^{l}V_{h}^{l}\right)\right)\right)$$

where $V_{h}^{l}$ denotes the value projections of the superpixel tokens. After several layers, the refined superpixel tokens are mapped back to the pixel grid. For pixel i, the restored graph feature is:

(10) $$Z_{g}\left(i\right)=T^{L}\left(g_{i}\right)$$

where $g_{i}$ denotes the superpixel index of pixel i, and $T^{L}$ is the final superpixel features. The output $Z_{g}$ is a dense feature map aligned with the original image. It contains graph-level context from neighboring regions and is later fused with the pixel-level representation from WP-ViT. Because the tokens correspond to superpixels, the attention weights in GS-ViT can also be visualized as region-level evidence for the model prediction.

### 3.3. Windowed Pixel Vision Transformer (WP-ViT)

WP-ViT is introduced to preserve dense pixel-level information that may be weakened by superpixel aggregation. Although GS-ViT provides compact region-level context, each superpixel token inevitably summarizes multiple pixels. This is useful for semantic consistency, but it can also smooth subtle spectral differences, narrow boundaries, and small land-cover structures. These details are important in hyperspectral classification, where different classes may have similar spatial appearances but distinct local spectral responses. A direct pixel-level transformer could model these variations, but global self-attention over all pixels is computationally expensive for full HSI scenes. WP-ViT therefore uses local window attention to retain pixel-level resolution while controlling the attention cost.

Instead of applying attention to all N pixels at once, WP-ViT partitions the feature map into local windows. To enable spatial–spectral interaction across adjacent windows while maintaining low computational cost, we adopt a shifted window mechanism. In layer l, the feature map of size H×W×C is partitioned into regular non-overlapping windows of size $S_{w}\times S_{h}$ (denoted as $\Omega_{r}$). In the subsequent layer l+1, the window partitioning is shifted by $\left([S_{w}/2],[S_{h}/2]\right)$ pixels from the top-left corner. For a window partition $\Omega_{r}$, self-attention is computed only among the pixels inside that window [19]. For the $h^{th}$ attention head at layer l, the attention matrix is represented as:

(11) $$P_{h,r}^{l}=softmax\left(\frac{Q_{h,r}^{l}{\left(K_{h,r}^{l}\right)}^{T}}{\sqrt{d_{h}}}\right)$$

where $Q_{h,r}^{l}$, $K_{h,r}^{l}$ and $V_{h,r}^{l}$ are the query, key, and value projections of the pixel tokens in window $\Omega_{r}$. The corresponding attention output is:

(12) $$E_{h,r}^{l}=P_{h,r}^{l}V_{h,r}^{l}$$

Each WP-ViT block follows a residual transformer structure. After window attention and MLP refinement, the update is then updated as:

(13) $$E^{l+1}=E^{l}+Proj\left(W_{Attn}\left(LN\left(E^{l}\right)\right)\right)+MLP\left(LN\left(E^{l}+Proj\left(W_{Attn}\left(LN\left(E^{l}\right)\right)\right)\right)\right)$$

In addition, WP-ViT employs a residual path to keep a spectral skip connection from the input latent feature vector to the output pixel representation. This skip path helps retain the original spectral evidence after local spatial attention, which summarized the final pixel-level representation as:

(14) $$Z_{p}=\phi_{out}\left(E^{L}\right)+\phi_{skip}\left(F_{p}\right)$$

where $E^{L}$ denotes the final pixel features, $\phi_{out}(\cdot )$ projects the transformer output to the fusion dimension, and $\phi_{skip}(\cdot )$ maps the original latent pixel features to the same dimension. The output $Z_{p}$ is a dense representation aligned with the original image grid. It complements the graph-contextual feature $Z_{g}$ from GS-ViT. WP-ViT emphasizes local spectral–spatial detail, while GS-ViT emphasizes region-level graph context. This separation is important because it prevents the model from relying only on coarse superpixel representations and gives the later fusion module access to both fine and contextual evidence.

### 3.4. Pixel–Graph Fusion and Classification (PG-FC)

The GS-ViT and WP-ViT branches provide different types of evidence. GS-ViT produces a graph-contextual representation in which each pixel receives information from its superpixel and neighboring regions. This is useful for region consistency and long-range spatial context. WP-ViT produces a dense pixel representation that preserves local spectral–spatial details and boundary information. Using either branch alone would leave part of the problem under-modeled, where a graph–superpixel branch may smooth local variations, whereas a pixel branch may lack explicit region-level structure. PG-FC is therefore used to combine the two transformer representations before prediction.

After the dense graph-contextual representation, $Z_{g}$ and the dense pixel representation $Z_{p}$ are produced by GS-ViT and WP-ViT, respectively. Since both are aligned with the original image grid, they can be fused pixel by pixel. The fusion module first estimates a soft gate from the concatenated features:

(15) $$G=\sigma \left(W_{f}\left[Z_{p},Z_{g}\right]+b_{f}\right)$$

where [⋅,⋅] denotes channel-wise concatenation, σ(⋅) is the sigmoid function, $W_{f}$ and $b_{f}$ are learnable parameters and bias, respectively. The gate controls how much graph–superpixel context should be added to each pixel representation. The fused transformer representation is then computed as:

(16) $$Z_{f}=f_{fuse}\left(Z_{p},Z_{g}\right)=\left[Z_{p},Z_{g},Z_{p}+G⊙Z_{g}\right]$$

This residual form keeps the dense pixel representation as the base feature and selectively injects graph-contextual information. It is useful for HSI classification because the need for graph context may vary across the image. The final classifier receives the pixel representation, the graph representation, and the fused representation, produces $\hat{Y}$ as shown in Equation (2).

During training, only labeled pixels are included in the objective. Let Y be the one-hot ground-truth label matrix and $M_{label}\in {\left\{0,1\right\}}^{N\times 1}$ be the binary label mask. The masked cross-entropy loss is derived as:

(17) $$L=-\sum_{i=1}^{N}\sum_{c=1}^{C}M_{lable,ic}Y_{ic}log{\hat{Y}}_{ic}$$

This objective follows the standard sparse-label setting in hyperspectral image classification, where only a small subset of pixels is used for supervision. The resulting prediction combines the local precision of WP-ViT and the region-level context of GS-ViT through adaptive pixel–graph transformer fusion.

### 3.5. Explainable Attention and Gradient Analysis (EAGA)

EAGA is designed to identify which spatial regions and spectral bands support the model prediction. This is important for hyperspectral image analysis because a class decision is often determined by both spatial context and wavelength-specific responses [1]. Although transformer attention can indicate interactions among tokens, attention alone does not directly quantify how the input spectrum affects a class score [33]. Therefore, the main explanation in EAGA is based on class-specific gradients with respect to the input HSI. These gradients are then organized into pixel-level relevance, superpixel-level relevance, and spectral-band importance.

For a target class c, let $s_{c}$ denote the class score used for explanation. The score may correspond to the response at a selected pixel or to an aggregated class response over a predicted class region. The sensitivity of this score to the input value at pixel j and spectral band b is defined as:

(18) $$G_{c}\left(j,b\right)=\left|\frac{\partial s_{c}}{\partial X_{j,b}}\right|$$

The absolute value is used because both positive and negative changes in the input can indicate that a spatial–spectral component influences the class decision. By summing the sensitivity over all spectral bands, the pixel-level class relevance is obtained as:

(19) $$R_{c}^{pixel}\left(j\right)=\sum_{b=1}^{B}G_{c}\left(j,b\right)$$

This relevance map indicates which image locations most affect the target class score.

Pixel-level gradients can be spatially fragmented, especially in hyperspectral scenes where neighboring pixels often belong to the same material. Since the proposed model reasons explicitly over superpixel regions, EAGA further aggregates pixel relevance within each superpixel. For the $m^{th}$ superpixel region $R_{m}$, the superpixel-level relevance can be expressed as:

(20) $$R_{c}^{sp}\left(m\right)=\frac{1}{\left|R_{m}\right|}\sum_{j\in R_{m}}R_{c}^{pixel}\left(j\right)$$

This is the main spatial explanation used in EAGA. It converts dense gradient sensitivity into region-level evidence aligned with the graph–superpixel representation. The region relevance can be restored to the image plane by assigning each pixel the relevance value of its corresponding superpixel:

(21) $${\tilde{R}}_{c}^{sp}\left(j\right)=R_{c}^{sp}\left(g_{j}\right)$$

where $g_{j}$ denotes the superpixel index of pixel j. This produces an explanation map whose spatial units are consistent with the GS-ViT tokens.

The same gradient tensor is also used to derive spectral-band importance. Instead of summing over bands, the sensitivity of each band is accumulated over all spatial positions:

(22) $$R_{c}^{band}\left(b\right)=\sum_{j=1}^{N}G_{c}\left(j,b\right)$$

This score measures how strongly the $b^{th}$ spectral channel contributes to the target class score across the image. To compare the relative contribution of different bands within the same class, the band relevance is computed:

(23) $${\bar{R}}_{c}^{band}\left(b\right)=\frac{R_{c}^{band}\left(b\right)}{\sum_{b^{\prime }=1}^{B}R_{c}^{band}\left(b^{\prime }\right)+\epsilon }$$

where ϵ denotes a small positive constant used to avoid division by zero. The ranked values of ${\bar{R}}_{c}^{band}(b)$ identify the wavelengths that are most influential for class c. This provides a spectral explanation that is directly tied to the physical nature of hyperspectral data.

Attention-based evidence is used as a complementary explanation. The attention flow in GS-ViT indicates how information is transferred among superpixel tokens, while graph-biased attention shows how adjacency-guided region interactions affect the prediction. These attention responses are projected back to the image grid through the superpixel assignment. Together, gradient relevance and attention evidence provide a multi-level explanation: pixel relevance describes local sensitivity, superpixel relevance describes region-level class evidence, spectral-band importance describes wavelength contribution, and attention maps describe graph–superpixel interaction.

## 4. Experiments

### 4.1. Datasets

Experiments are conducted on four widely used hyperspectral image classification benchmarks: Indian Pines, Pavia University, Salinas [34], and WHU-Hi-LongKou [35]. These datasets cover different land-cover distributions and scene structures, allowing the proposed method to be evaluated under both agricultural and urban conditions. Indiana Pines contains agricultural categories with strong class imbalance and high inter-class spectral similarity. Pavia University represents an urban scene with classes such as asphalt, meadows, gravel, trees, bare soil, and shadows. Salinas is an agricultural scene with larger homogeneous crop regions and several spectrally similar vegetation categories.

To further validate the robustness and generalization of ESG-ViT, we evaluate its performance on a modern, high-resolution UAV-borne HSI dataset: WHU-Hi-LongKou [35], acquired by a Headwall Nano-Hyperspec sensor over an agricultural scene with nine land-cover categories.

The Indian Pines image has a spatial size of 145×145, with 200 spectral bands after removing water absorption bands, and contains 16 land-cover classes. The Pavia University image has a spatial size of 610×340, with one hundred and three spectral bands and nine classes. The Salinas image has a spatial size of 512×217, with 204 spectral bands and 16 classes. Background pixels are excluded from model training and evaluation. The WHU-Hi-LongKou image has a spatial size of 550×400, with two hundred and seventy spectral bands, and contains nine land-cover classes. Background pixels are excluded from model training and evaluation. The class-wise sample information and the training, validation, and test splits are reported separately for the four datasets in Table 1, Table 2, Table 3 and Table 4.

### 4.2. Experimental Settings

Each hyperspectral cube is standardized band-wise before being input to the network. Training samples are selected class by class according to the specified training ratio. For Indian Pines, Pavia University, and Salinas, the default training sample ratios are set to 1%, 0.57%, and 0.48%, respectively, with 1% of non-background pixels sampled as the validation set and the rest used for testing. For WHU-Hi-LongKou, a fixed split of 100 labeled samples per category is randomly selected for training and validation, as outlined in Table 4. Each experiment is repeated over five random seeds, and the final quantitative results are reported as mean ± standard deviation.

The proposed model is optimized using Adam with a learning rate of $5\times 10^{-4}$. The maximum number of training epochs is set to 600, and the model checkpoint with the lowest validation loss is selected for final testing. Superpixels are generated before network training using the adopted LDA-SLIC-style preprocessing strategy [7]. Unless otherwise stated, the superpixel scale is set to 100. The spectral transformation stem maps the input HSI into 128 latent channels. The GS-ViT and WP-ViT branches use 64-dimensional transformer embeddings in the main configuration, and WP-ViT uses local window attention for efficient dense pixel modeling.

The proposed model is optimized using the Adam optimizer with an initial learning rate of $5\times 10^{-4}$, weight decay of $10^{-4}$. The maximum number of training epochs is set to 600, and the model checkpoint with the lowest validation loss is selected for final testing. Superpixels are generated before network training using the adopted LDA-SLIC-style preprocessing strategy [7]. Unless otherwise stated, the superpixel scale is set to 100. The spectral transformation stem maps the input HSI into 128 latent channels (C=128). The GS-ViT branch consists of $L_{gs}=4$ graph transformer blocks with embedding dimension D=64 and $H_{gs}=4$ attention heads. The WP-ViT branch comprises $L_{wp}=4$ windowed transformer blocks with the same embedding dimension D=64 and $H_{wp}=4$ attention heads, using a local window size of $S_{w}\times S_{h}=7\times 7$. All experiments are conducted on an NVIDIA GeForce RTX 4080 GPU with 16 GB of VRAM under the PyTorch 1.11 framework.

The evaluation metrics include overall accuracy (OA), average accuracy (AA), Cohen’s kappa coefficient, and per-class accuracy. OA measures the percentage of correctly classified test pixels, while AA computes the mean accuracy over all classes and is more sensitive to class imbalance. Cohen’s kappa evaluates the agreement between prediction and ground truth after accounting for chance agreement. In addition to quantitative metrics, explanation maps are used to evaluate spatial consistency, boundary preservation, and interpretability.

### 4.3. Overall Comparison

The proposed method is compared with DBDA [36], DBMA [37], FDSSC [38], SSRN [39], CEGCN [7], FDGCN [40], WFCG [41], and CEGAT [8] on Indian Pines, Salinas, and Pavia University; SVM, FNEA-OO [42], SVRFMC [43], CNNCRF [35] on WHU-Hi-LongKou dataset. SSFTT [21] and SpectralFormer [20] are compared as transformer-based baselines. The detailed class-wise accuracies and summary metrics are reported in Table 5, Table 6, Table 7 and Table 8. The evaluation uses overall accuracy (OA), average accuracy (AA), and Cohen’s kappa coefficient. The value in parentheses denotes the standard deviation over repeated runs.

For the Indian Pines dataset (Table 5), which is characterized by severe class imbalance and high spectral similarity, the proposed ESG-ViT achieves the highest overall accuracy (OA) of 99.10%, average accuracy (AA) of 98.75%, and a kappa coefficient of 98.97%. Compared to the strongest competing traditional baseline, CEGAT, the proposed method yields improvements of 0.96% in OA, 0.55% in AA, and 1.09% in kappa. Notably, the performance gap is even wider when compared to the newly integrated HSI Transformer baselines: ESG-ViT outperforms SSFTT (97.61% OA) and SpectralFormer (68.49% OA) by 1.49% and 30.61%, respectively. The class-wise metrics further reveal that the proposed model secures the best classification accuracy across most categories, demonstrating the effectiveness of the dual-branch architecture under constrained training sizes.

Table 6 presents the classification performance on the Salinas dataset. Here, the proposed model obtains 99.23% OA, 99.66% AA, and 99.15% kappa, outperforming all compared methods and establishing the most balanced land-cover classification map. Relative to CEGAT, the strongest competing traditional baseline, the proposed method enhances OA from 99.18% to 99.23%, AA from 99.47% to 99.66%, and kappa from 99.08% to 99.15%. Crucially, ESG-ViT maintains a clear superiority over the transformer baselines SSFTT (98.32% OA) and SpectralFormer (82.78% OA). While classification on Salinas is often close to saturation, the graph–superpixel context in ESG-ViT successfully stabilizes predictions across large homogeneous crop regions, while the parallel pixel branch retains the discriminative local information necessary to separate fine classes.

On the urban scene of the Pavia University dataset (Table 7), the proposed method secures the leading position with 99.33% OA, 98.94% AA, and 99.03% kappa. This represents a steady improvement over the strongest competing baseline, CEGAT (99.20% OA, 98.83% AA, 98.90% kappa), and substantially surpasses the HSI transformer models SSFTT (97.15% OA) and SpectralFormer (78.70% OA). Class-wise evaluation highlights that the model achieves perfect accuracy (100%) on classes 2, 5, and 6, and delivers the highest performance on classes 7 and 8. These results support the merit of combining region-level graph reasoning and dense pixel-level modeling in urban environments containing sharp geometric boundaries and mixed materials.

Finally, the model’s scalability was validated on the high-resolution UAV-borne WHU-Hi-LongKou dataset (Table 8). ESG-ViT achieves an outstanding OA of 99.71%, AA of 99.07%, and kappa of 99.62%, consistently outperforming all reference methods. Specifically, compared with its closest competitor, SSFTT, the proposed method yields improvements of 0.30% in OA and 0.39% in kappa, while outperforming CNNCRF and SpectralFormer by 0.80% and 3.04% in OA. The results confirm that embedding the superpixel graph topology as a bias in the self-attention matrix effectively preserves crop boundaries in fine-scale agricultural mapping.

It is worth noting that the performance of SpectralFormer, a pure spectral–spatial transformer baseline, is significantly lower than that of SSFTT and the proposed ESG-ViT across the datasets. This disparity highlights a fundamental limitation of pure Transformer architectures in hyperspectral image classification: the lack of local inductive biases. Unlike standard vision tasks with massive pre-training datasets, HSI classification frequently suffers from extremely limited training samples. When trained from scratch, a pure self-attention mechanism struggles to learn local spatial correlations and is highly prone to overfitting. In contrast, SSFTT mitigates this by incorporating a convolutional (3D/2D CNN) stem to extract local spatial–spectral features prior to the transformer blocks. The proposed ESG-ViT goes a step further by directly embedding the superpixel graph topology as a structural bias in the self-attention matrix. The superior performance of SSFTT and ESG-ViT over SpectralFormer demonstrates that incorporating spatial structural priors (either grid-based convolutions or irregular graph topologies) is crucial for Transformer-based architectures to generalize effectively under small-sample constraints.

Overall, the proposed method consistently achieves the best classification metrics across all four HSI benchmarks. While the performance gain is particularly prominent on more challenging datasets like Indian Pines and WHU-Hi-LongKou, it remains robust and stable on Salinas and Pavia University. These experimental findings validate the core design of ESG-ViT: the GS-ViT branch ensures global, graph-level regional consistency, the WP-ViT branch preserves local, pixel-level details, and their joint fusion provides robust, edge-preserving classification in diverse remote sensing environments.

### 4.4. Ablation Studies

To systematically evaluate the contribution and necessity of each branch in the dual-branch ESG-ViT architecture, we conduct detailed model ablation experiments on the Salinas dataset under a standard training ratio of 0.48%. Specifically, we compare three distinct configurations: (1) the GS-ViT branch only, (2) the WP-ViT branch only, and (3) the complete dual-branch ESG-ViT model.

The experimental results in Table 9 demonstrate that the complete ESG-ViT achieves the highest performance with an OA of 99.23% ± 0.30%. Disabling the GS-ViT branch (WP-ViT only) results in a classification performance of 96.84% ± 0.40% OA, representing a significant accuracy drop of 2.39%. This occurs because, without the GS-ViT branch, the model loses the ability to capture region-level spatial–spectral consistency and long-range topological relationships over irregular land-cover structures, relying solely on local grid-based windows.

Conversely, disabling the WP-ViT branch (GS-ViT only) leads to a larger performance degradation of 3.88%. This more severe drop indicates that while the superpixel-level representation is efficient for global context, the spatial aggregation/pooling step inevitably smooths out fine spatial details, narrow crop boundaries, and sub-superpixel spectral variations. Without the dense pixel-level modeling of WP-ViT to preserve local boundaries and details, the model’s discriminative capability is heavily compromised. Therefore, the synergy between the two branches is essential: the GS-ViT branch ensures global and regional structural consistency, while the WP-ViT branch preserves fine spatial–spectral boundaries, and their dynamic fusion via the PG-FC gate yields optimal HSI classification.

In the proposed framework, the scale parameter S of the LDA-SLIC algorithm controls the initial size and total number of segmented superpixels (M), which determines the graph topology of the GS-ViT branch. To evaluate the sensitivity and robustness of ESG-ViT to this hyperparameter, we analyze the model’s classification performance under scale parameters of 50, 100, 150, and 200 on the Salinas dataset. Table 9 reports the overall accuracy (OA), average accuracy (AA), and kappa coefficient for each scale. As shown in Table 10, the classification performance of ESG-ViT remains highly stable across the entire range of scale values. The overall accuracy varies from a peak of 99.23% ± 0.29% at scale 100 to 99.18% ± 0.14% at scale 200, representing an extremely narrow fluctuation margin of only 0.05%. The average accuracy (AA) and kappa coefficient exhibit similar robustness, consistently staying above 99.40% and 99.00%, respectively.

This parameter stability can be attributed to two main factors: (1) the dual-branch complementary architecture, where the pixel-level branch (WP-ViT) operates independently of the superpixel scale and acts as a “safety net” to capture fine boundary features even when the superpixels are partitioned too coarsely; and (2) the adaptive fusion gate in the PG-FC module, which dynamically regulates the information blend between the two branches pixel-by-pixel. Consequently, if the superpixel scale is set sub-optimally, the fusion module automatically increases the attention weight of the pixel-level branch to compensate for the loss of detail. This structural self-regulation reduces the need for extensive hyperparameter tuning during real-world deployments.

### 4.5. Computational Cost and Efficiency Evaluation

To evaluate the practical efficiency and resource trade-offs of ESG-ViT, we conduct a comprehensive empirical benchmark on the Salinas dataset. Table 11 presents the empirical measurement results, including trainable parameter count (Params, M), floating-point operations (FLOPs, G), training time per epoch (seconds), test inference time per full scene (seconds), peak GPU memory footprint (VRAM, GB), and OA.

As presented in Table 11, standard Vision Transformers like SpectralFormer [20] suffer from immense computational overhead, requiring 6748.489 G FLOPs and taking 2.8414 s per scene for test inference. Similarly, SSFTT [21] requires 1164.069 G FLOPs and 0.8579 s per scene. In sharp contrast, the proposed ESG-ViT requires only 231.301 G FLOPs, representing a 29.2-time reduction in FLOPs compared to SpectralFormer and a five-time reduction compared to SSFTT, while completing full-scene test inference in just 0.0412 s (over 68 times faster than SpectralFormer and 20 times faster than SSFTT).

Furthermore, Table 11 incorporates branch ablation baselines (GS-ViT Only and WP-ViT Only). GS-ViT Only requires 0.815 M parameters and 138.539 G FLOPs with 95.35% OA, whereas WP-ViT Only requires 0.168 M parameters and 76.712 G FLOPs with 96.84% OA. Fusing both branches into ESG-ViT (1.137 M parameters, 231.301 G FLOPs) boosts classification accuracy to 99.23% OA, demonstrating that the dual-branch integration yields substantial performance gains with negligible inference latency (0.0412 s). As emphasized by De Lucia et al. [32], reducing computational complexity and inference latency while retaining explainability is critical for onboard hyperspectral analysis in resource-constrained edge computing environments.

### 4.6. Explainability Analysis

The explainability analysis evaluates whether the proposed ESG-ViT framework provides meaningful spatial and spectral evidence for its predictions. Hyperspectral image classification depends on both where a material appears (spatial layout) and which spectral channels distinguish it from other classes (spectral signatures). Therefore, our gradient-based Explainable Attention and Gradient Analysis (EAGA) module generates visualizations from two complementary perspectives: (1) region-level spatial explanation, where class-specific gradients are aggregated onto superpixels, and (2) spectral channel importance, which displays a class-wise importance heatmap across the hyperspectral bands. To contextualize the spectral channel analysis, it is important to note that the hyperspectral datasets used in our experiments were acquired by different imaging sensors with distinct physical designs: (1) the Indian Pines and Salinas datasets were captured by the Airborne Visible/Infrared Imaging Spectrometer (AVIRIS) sensor [44], which features 224 spectral bands spanning 400 nm to 2500 nm; (2) the Pavia University dataset was acquired by the Reflective Optics System Imaging Spectrometer (ROSIS-03) sensor [45], recording 115 bands across 430 nm to 860 nm; and (3) the WHU-Hi-LongKou dataset was gathered via a UAV-borne Headwall Nano-Hyperspec sensor [35], which collects 270 channels in the visible and near-infrared range (400 nm to 1000 nm). The preprocessing stage for each dataset removes noisy and atmospheric water absorption bands, resulting in 200, 204, 103, and 270 channels for Indian Pines, Salinas, Pavia University, and WHU-Hi-LongKou, respectively.

#### 4.6.1. Indian Pines Dataset

**Figure 3:**
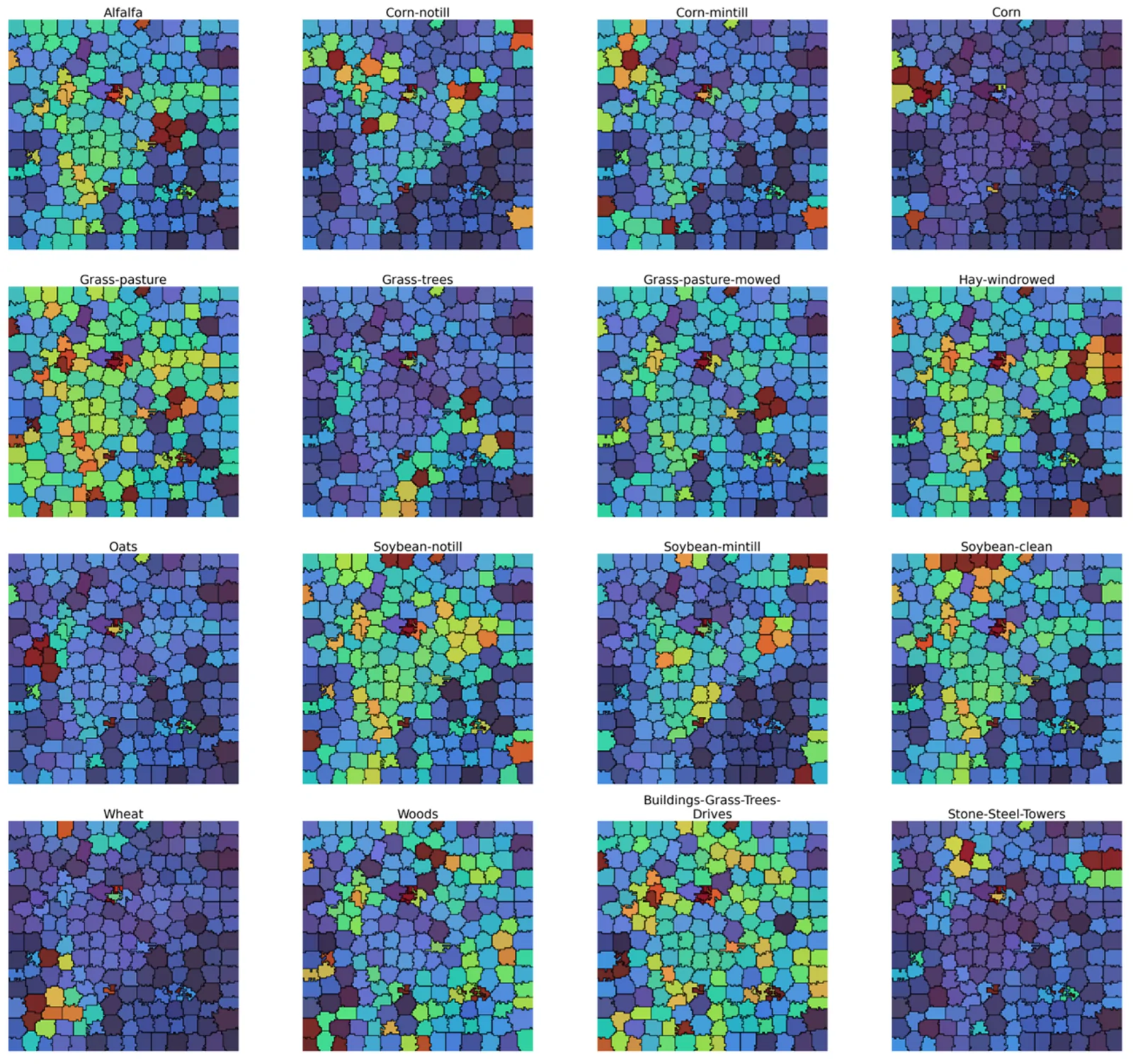
Class-specific spatial superpixel importance on Indian Pines dataset. The heatmap colors indicate the relative importance of superpixels, with warmer colors representing higher importance and cooler colors representing lower importance.

On the Indian Pines dataset, the spatial importance maps (Figure 3) demonstrate that the model successfully concentrates its gradients within the ground-truth boundaries of agricultural fields. For instance, Corn-notill (Class 2) and Soybean-notill (Class 10), which are represented as regular rectangular blocks in the scene, show highly concentrated importance values within their respective field boundaries, showing no leakage into adjacent fields of different categories. This spatial alignment demonstrates that the superpixel-level aggregation effectively suppresses pixel-level noise, mapping class relevance directly to meaningful spatial regions.

**Figure 4:**
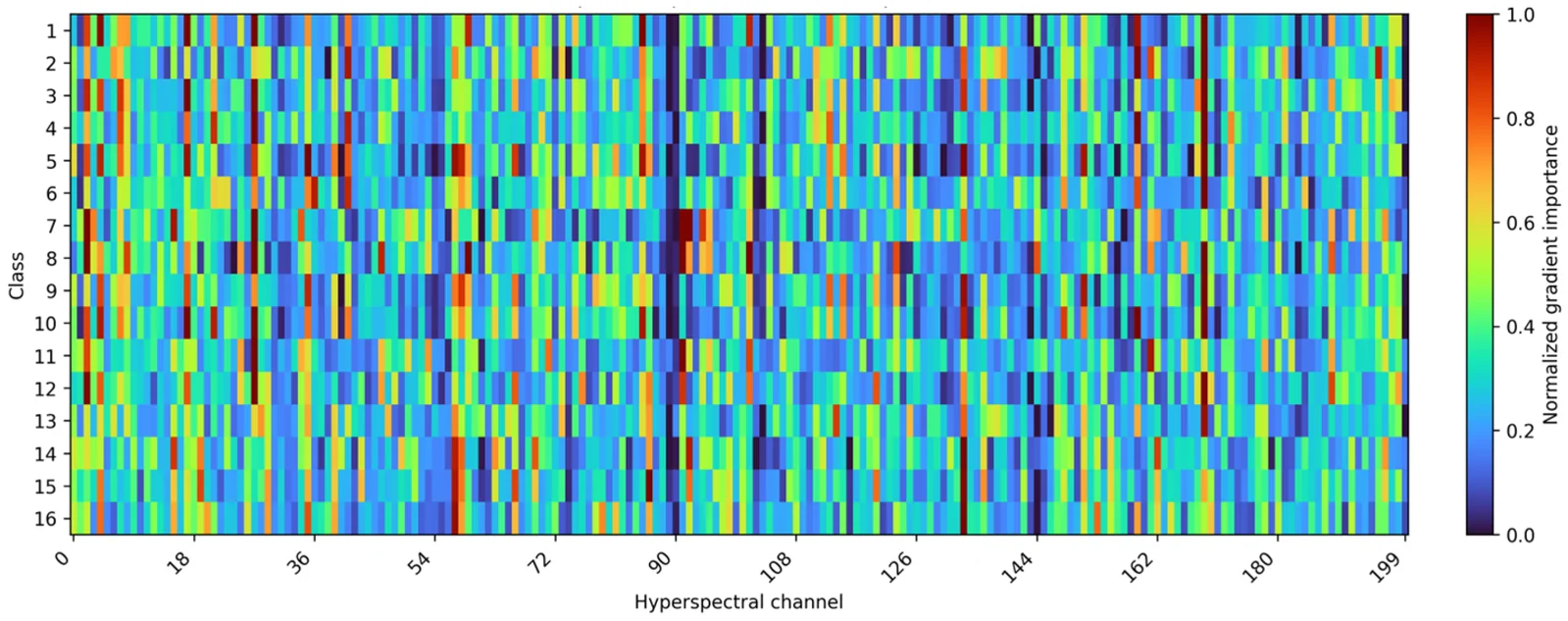
Class-specific spectral channel importance on Indian Pines dataset.

Spectrally, the channel importance heatmap (Figure 4) provides a macro-level physical explanation of band contribution. Instead of showing scattered, arbitrary band sensitivities, the model exhibits high physical consistency: around channel 90, all categories show complete insensitivity. This region corresponds directly to the strong atmospheric water vapor absorption bands (specifically AVIRIS bands 104–108), where the signal is heavily attenuated and noisy; the model correctly learns to ignore these zero-signal channels. Furthermore, around channel 144, classes 13 to 16 (Wheat, Woods, Buildings-Grass-Trees-Drives, and Stone-Steel-Towers) exhibit a highly pronounced, localized insensitivity, whereas crop classes remain active, suggesting that the model’s feature representation aligns with the distinct spectral profiles of man-made and non-vegetated materials.

#### 4.6.2. Salinas Dataset

**Figure 5:**
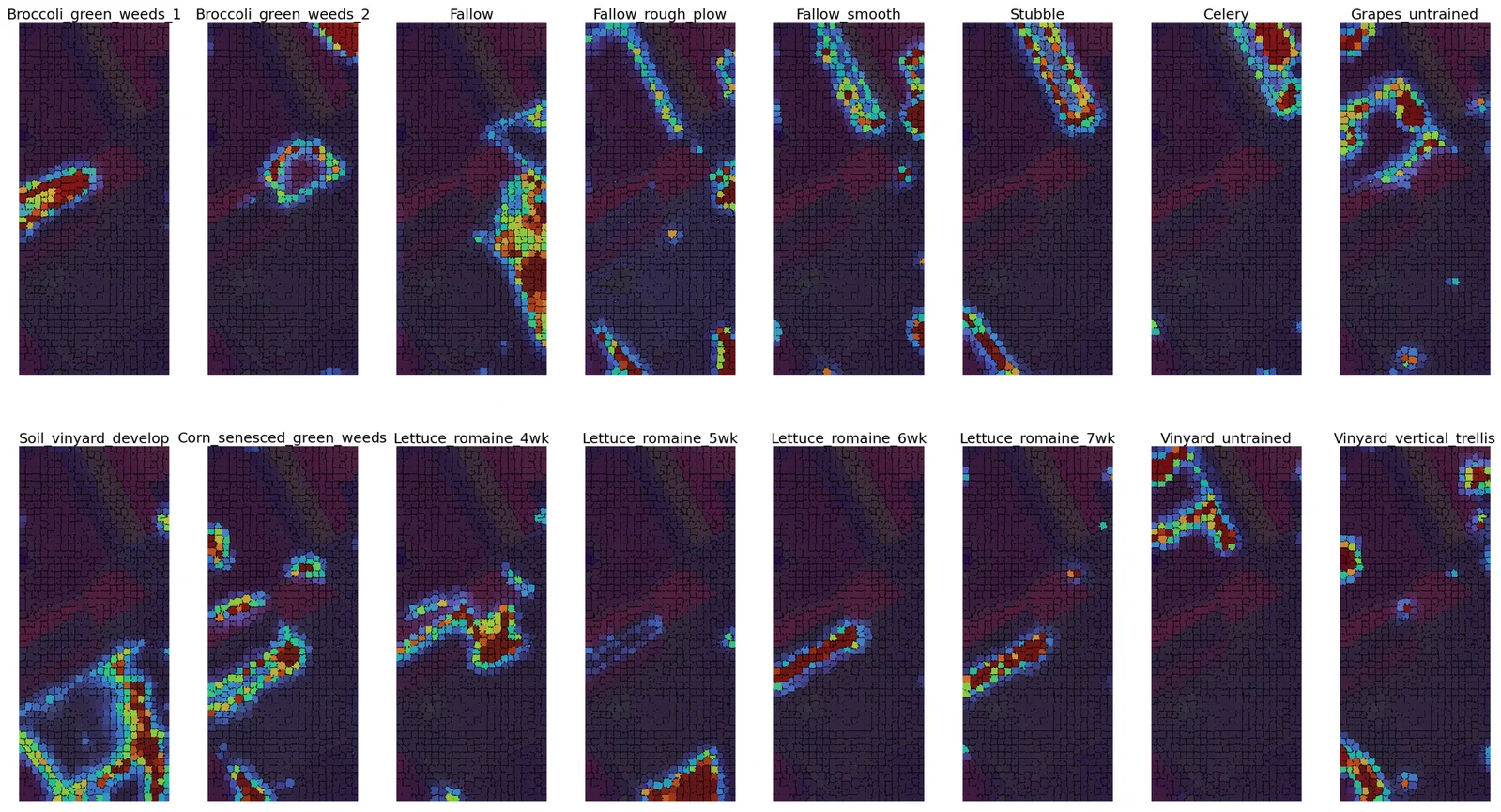
Class-Specific Spatial Superpixel Importance on Salinas Dataset. The heatmap colors indicate the relative importance of superpixels, with warmer colors representing higher importance and cooler colors representing lower importance.

On the Salinas dataset, which contains large, continuous, and homogeneous crop fields, the spatial relevance maps (Figure 5) highlight the entire fields with high internal homogeneity. For Fallow (Class 3) and Vineyard untrained (Class 15), the highlighted superpixels align precisely with their large-scale crop boundaries. This visual uniformity confirms that the GS-ViT branch successfully propagates spatial–spectral context across irregular superpixel graph nodes, ensuring that agricultural regions are processed as cohesive semantic units.

**Figure 6:**
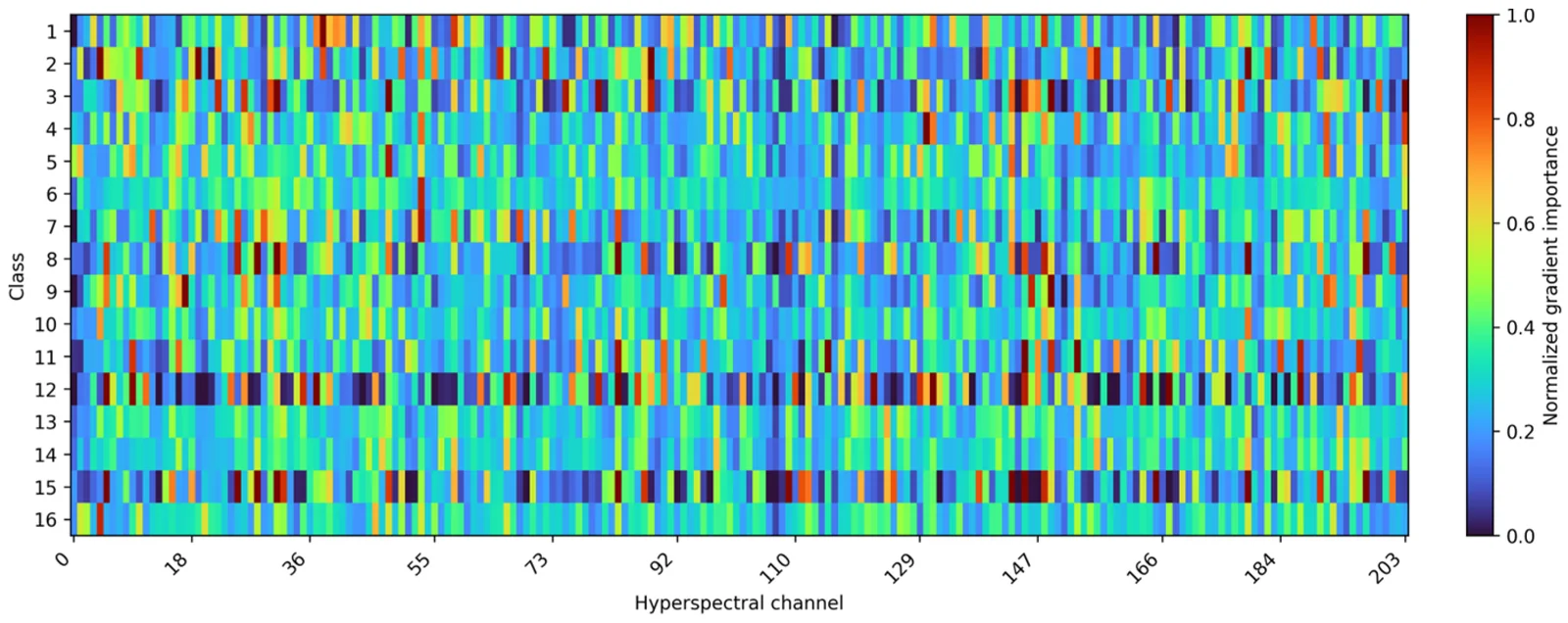
Class-Specific Spectral Channel Importance on Salinas Dataset.

In the spectral domain (Figure 6), the model shows a strong suppression of boundary and noise bands. All classes exhibit near-zero sensitivity around channel 1 and channel 110. The suppression at channel 1 likely represents sensor boundary noise (edge effect), while the band near channel 110 corresponds to the water vapor absorption bands, which are heavily impacted by atmospheric noise and attenuation, thus offering limited discriminative information. In contrast, Class 12 (Lettuce_romaine_5wk) and Class 15 (Vineyard untrained) show highly distinct sensitivity intervals compared to other classes, which is consistent with their characteristic spectral reflectance profiles and crop properties.

#### 4.6.3. Pavia University Dataset

**Figure 7:**
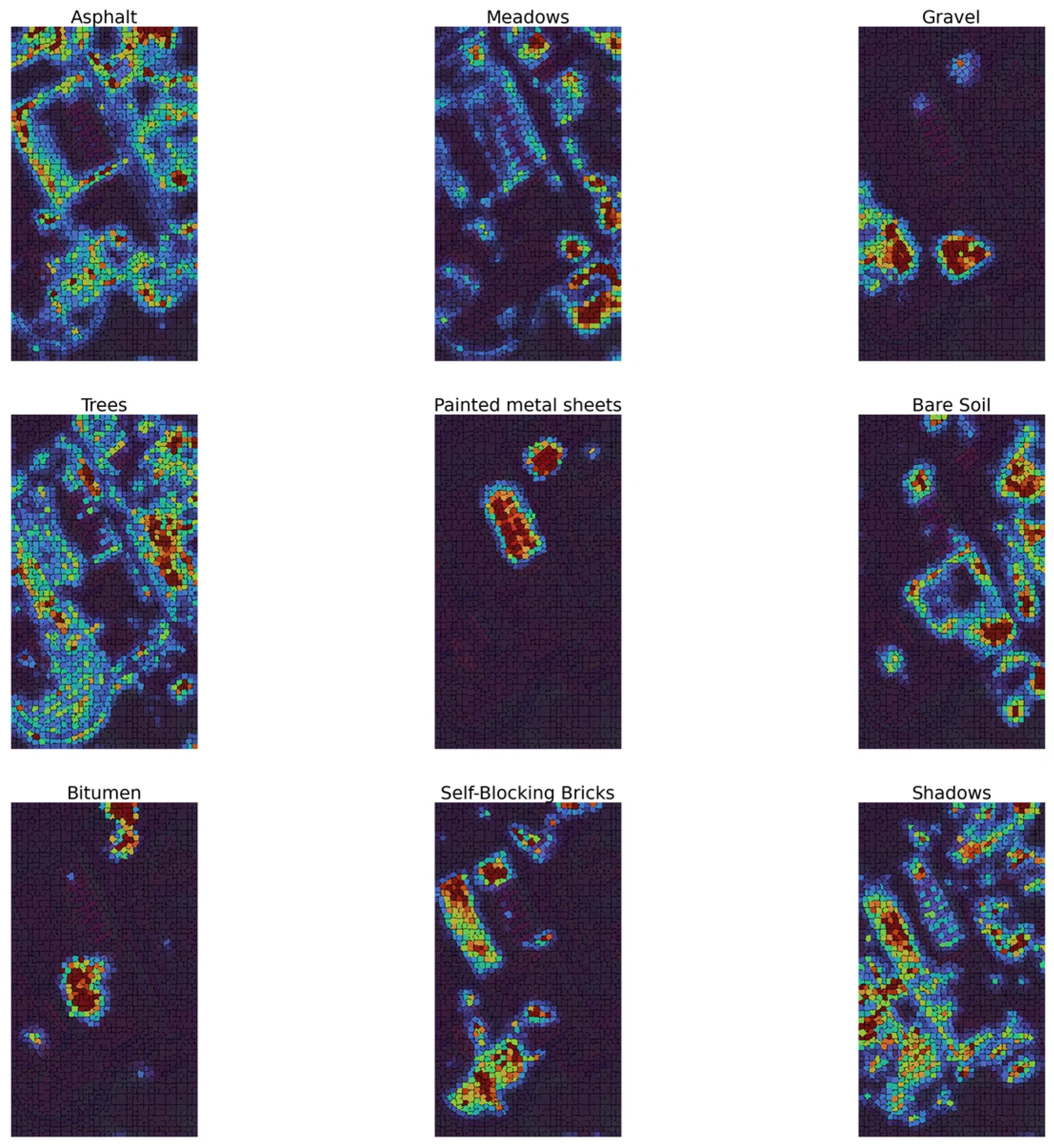
Class-specific spatial superpixel importance on Pavia University dataset. The heatmap colors indicate the relative importance of superpixels, with warmer colors representing higher importance and cooler colors representing lower importance.

The Pavia University dataset represents a complex urban scene containing narrow roads and irregular roofs. The spatial relevance maps (Figure 7) illustrate that the model accurately delineates thin and adjacent urban structures. For instance, Asphalt (Class 1) and Bitumen (Class 7) are sharply distinguished, with the vertical and horizontal asphalt road structures highlighted for Class 1, and bitumen roofs highlighted for Class 7. This sharp spatial delineation confirms that the windowed pixel-level branch (WP-ViT) prevents regional superpixel features from over-smoothing boundaries in fine-scale urban environments

**Figure 8:**
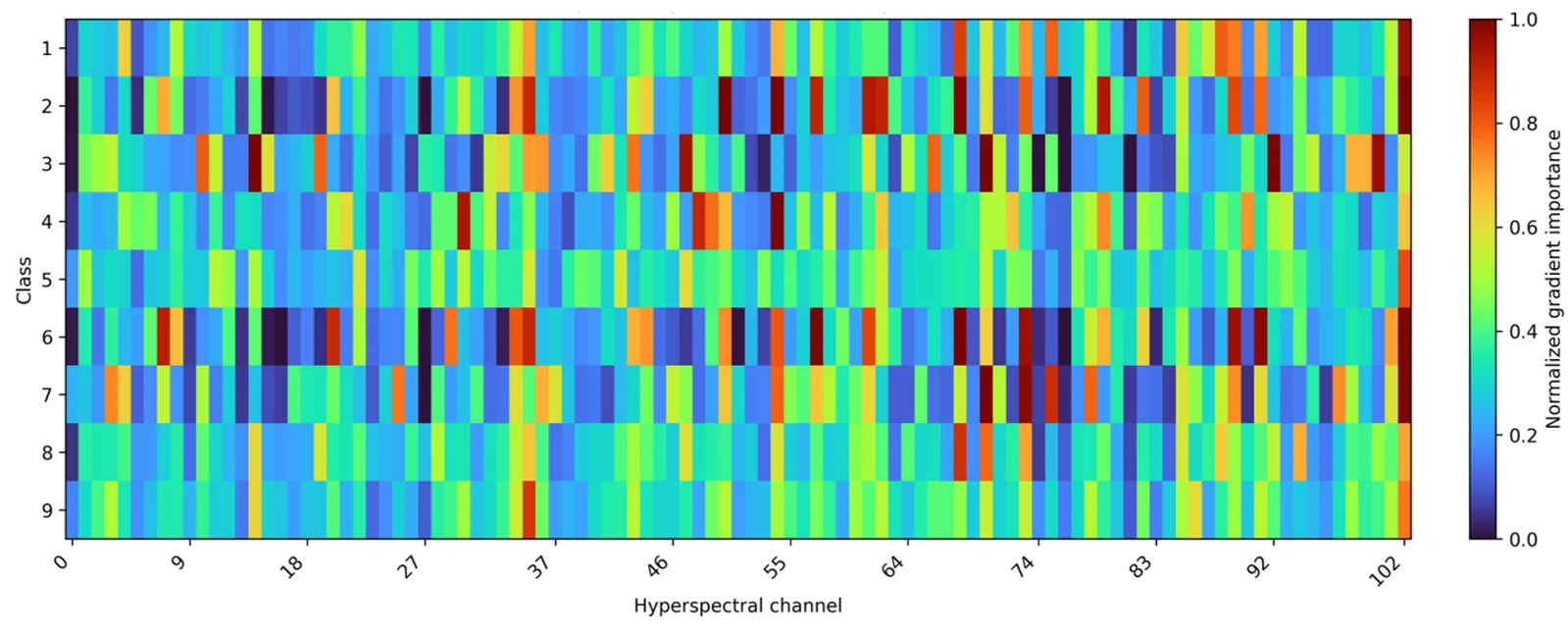
Class-specific spectral channel importance on Pavia University dataset.

Spectrally (Figure 8), all classes show near-zero sensitivity around channel 1 and channel 75. The low sensitivity at channel 1 is consistent with the edge effect of the ROSIS-03 sensor, while the low response around channel 75 may stem from sensor-level response variations or noise bands in this region. Interestingly, the very last channel exhibits high sensitivity across several urban classes, suggesting that this boundary band captures critical spectral information for separating artificial materials. Furthermore, Class 3 (Gravel) and Class 6 (Bare Soil) exhibit highly distinct sensitivity intervals compared to other vegetation-dominated classes (such as Meadows and Trees), reflecting their non-vegetated mineral composition.

#### 4.6.4. WHU-Hi-LongKou Dataset

**Figure 9:**
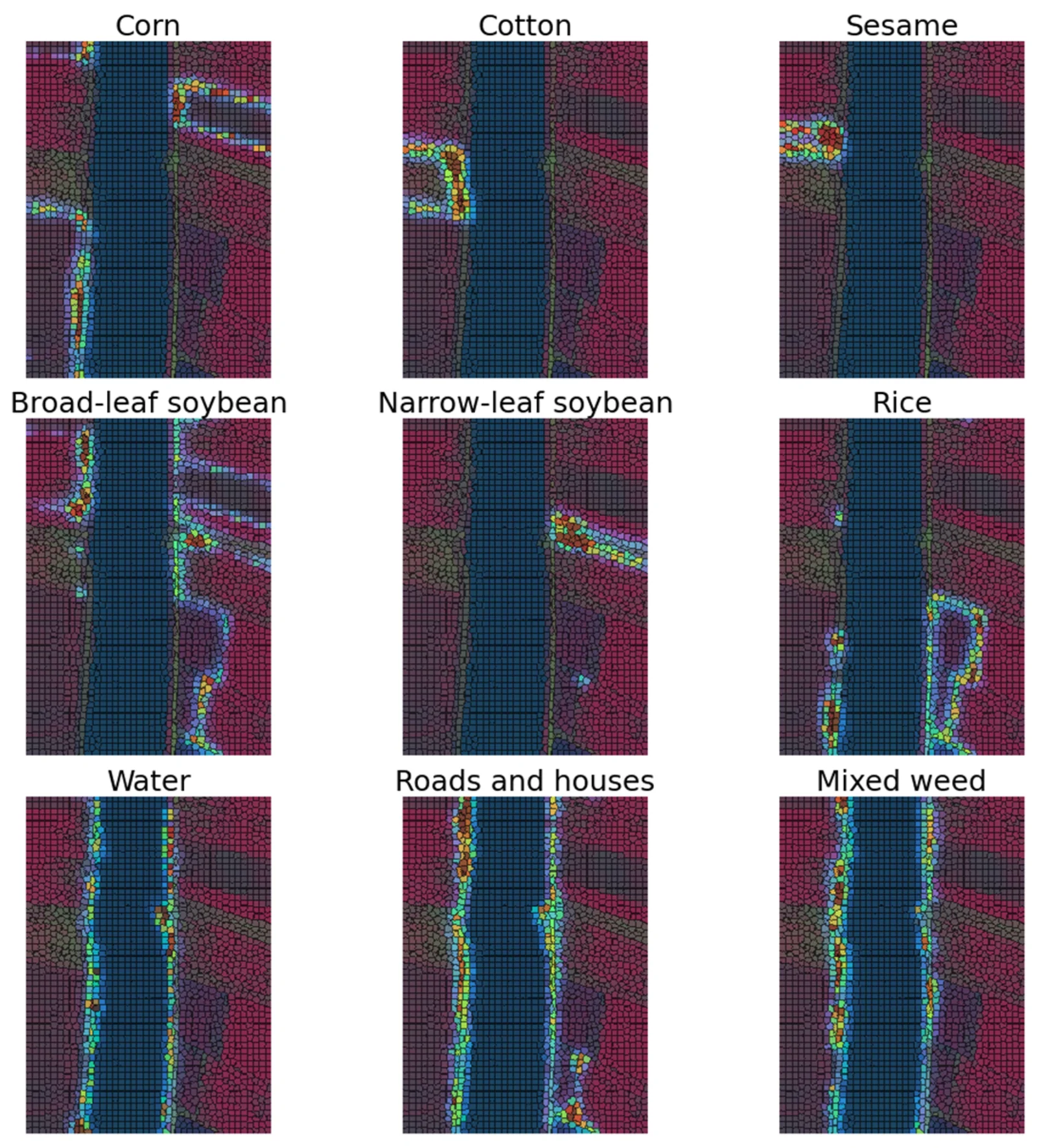
Class-specific spatial superpixel importance on WHU-Hi-LongKou dataset. The heatmap colors indicate the relative importance of superpixels, with warmer colors representing higher importance and cooler colors representing lower importance.

On the high-resolution UAV-borne WHU-Hi-LongKou dataset, the spatial relevance maps (Figure 9) reveal a distinct edge-sensitivity characteristic. Rather than highlighting the uniform interior regions of the classes, the high-gradient superpixels (colored in red and yellow) are concentrated along the spatial transition boundaries (edges) of each category. For example, in the Water category (Class 7), the highlighted regions are located strictly along the canal banks, while the uniform center of the river remains unhighlighted. A similar pattern is observed for crops like Corn (Class 1) and Rice (Class 6), where the outline boundaries of the agricultural plots are highlighted. This indicates that the model’s EAGA gradients are highly sensitive to spatial boundaries where the contrast is highest, representing a limitation in illustrating internal uniformity, but confirming the model’s reliance on edge-preserving details

**Figure 10:**
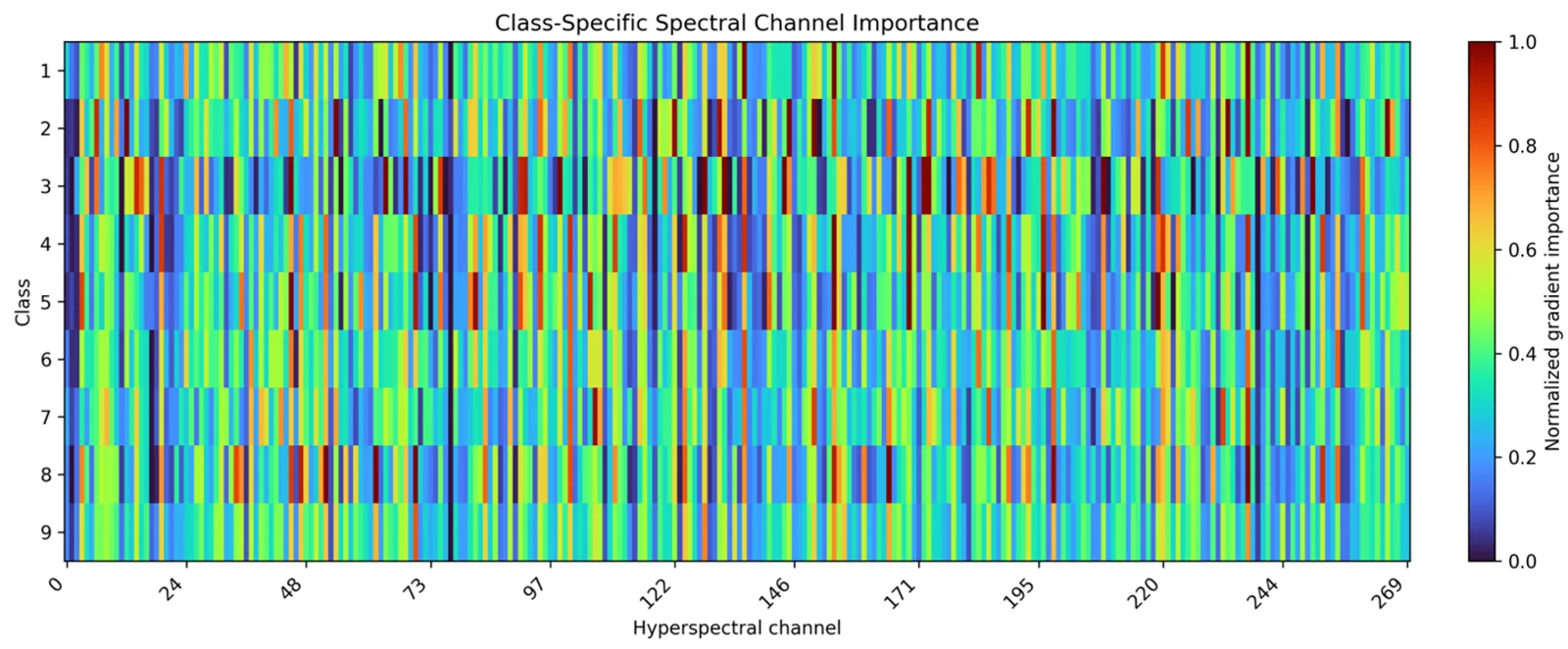
Class-specific spectral channel importance on WHU-Hi-LongKou dataset.

In the spectral domain (Figure 10), the first few channels exhibit near-zero sensitivity across all classes, indicating the model suppresses sensor edge noise. More importantly, two distinct vertical stripes of complete insensitivity are observed around channel 73 and channel 244 for all classes. These regions likely align with noisy channels or spectral response gaps typical of UAV-borne hyperspectral sensors, where the signal contributions are naturally minimized during preprocessing. Meanwhile, Class 3 (Sesame), Class 4 (Broad-leaf soybean), and Class 8 (Roads and houses) show prominent, widespread high sensitivity across many channels. This broad spectral response suggests that the model coordinates visible pigment bands and multiple NIR bands to successfully distinguish these classes from nearby vegetated and man-made backgrounds.

#### 4.6.5. Discussion on Explainability and Interpretation

Overall, these visual explanations across all four HSI benchmarks confirm that the proposed ESG-ViT does not generate fragmented, pixel-level noise, nor does it rely on arbitrary global features. The spatial relevance maps are highly coherent, showing a strong alignment with the irregular land-cover boundaries. Meanwhile, the spectral channel heatmaps demonstrate that the model automatically learns physical spectral properties (such as vegetation red-edges, chlorophyll absorption bands, and water absorption characteristics) from the data. This dual-level explainability provides a reliable, physically grounded verification of the model’s decision-making process, making ESG-ViT a robust and transparent tool for remote sensing HSI classification.

## 5. Conclusions

This paper proposed an Explainable Superpixel-Guided Graph Vision Transformer (ESG-ViT) for hyperspectral image analysis. The proposed framework combines two complementary transformer branches. GS-ViT represents irregular land-cover regions as superpixel tokens and incorporates graph adjacency information directly into self-attention. WP-ViT preserves dense local information through windowed pixel-level attention. Their adaptive fusion enables the model to use region-level context without losing important local spectral–spatial details.

ESG-ViT also provides explanations from spatial and spectral perspectives. Class-specific gradient responses are aggregated within superpixels to generate coherent region relevance maps, while channel-wise aggregation produces spectral importance heatmaps for different categories. The visualizations show that the model emphasizes relevant spatial regions and uses distinct spectral channels for different classes. Experiments on the Indian Pines, Salinas, and Pavia University datasets demonstrate that ESG-ViT consistently achieves strong classification performance compared with representative methods.

## Figures and Tables

**Table 1:** Category information of the Indiana Pines dataset.

Class	Category	Total	Training	Validation	Test
1	Alfalfa	46	1	1	44
2	Corn-notill	1428	15	12	1401
3	Corn-mintill	830	9	8	813
4	Corn	237	3	3	231
5	Grass-pasture	483	5	5	473
6	Grass-trees	730	8	8	714
7	Grass-pasture-mowed	28	1	1	26
8	Hay-windrowed	478	5	7	466
9	Oats	20	1	1	18
10	Soybean-notill	972	10	7	955
11	Soybean-mintill	2455	25	20	2410
12	Soybean-clean	593	6	8	579
13	Wheat	205	3	2	200
14	Woods	1265	13	14	1238
15	Buildings-grass-trees-drives	386	4	5	377
16	Stone-steel-towers	93	1	1	91
	Total	10,249	110	102	10,037

**Table 2:** Category information of the Pavia University dataset.

Class	Category	Total	Training	Validation	Test
1	Asphalt	6631	38	57	6536
2	Meadows	18,649	107	200	18,342
3	Gravel	2099	12	18	2069
4	Trees	3064	18	30	3016
5	Painted metal sheets	1345	8	13	1324
6	Bare soil	5029	29	54	4946
7	Bitumen	1330	8	13	1309
8	Self-blocking bricks	3682	21	32	3629
9	Shadows	947	6	10	931
	Total	42,776	247	427	42,102

**Table 3:** Category information of the Salinas dataset.

Class	Category	Total	Training	Validation	Test
1	Broccoli_green_weeds_1	2009	10	31	1968
2	Broccoli_green_weeds_2	3726	18	29	3679
3	Fallow	1976	10	22	1944
4	Fallow_rough_plow	1394	7	11	1376
5	Fallow_smooth	2678	13	27	2638
6	Stubble	3959	20	32	3907
7	Celery	3579	18	47	3514
8	Grapes_untrained	11,271	55	114	11,102
9	Soil_vinyard_develop	6203	30	54	6119
10	Corn_senesced_green_weeds	3278	16	32	3230
11	Lettuce_romaine_4wk	1068	6	10	1052
12	Lettuce_romaine_5wk	1927	10	14	1903
13	Lettuce_romaine_6wk	916	5	8	903
14	Lettuce_romaine_7wk	1070	6	9	1055
15	Vinyard_untrained	7268	35	76	7157
16	Vinyard_vertical_trellis	1807	9	25	1773
	Total	54,129	268	541	53,320

**Table 4:** Category information of the WHU-Hi-LongKou dataset.

Class	Category	Total	Training	Validation	Test
1	Corn	34,511	100	100	34,311
2	Cotton	8374	100	100	8174
3	Sesame	3031	100	100	2831
4	Broad-leaf soybean	63,212	100	100	63,012
5	Narrow-leaf soybean	4151	100	100	3951
6	Rice	11,854	100	100	11,654
7	Water	67,056	100	100	66,856
8	Roads and houses	7124	100	100	6924
9	Mixed weed	5229	100	100	5029
	Total	204,542	900	900	202,742

**Table 5:** Individual Class, OA, AA, and KPP of all methods on the Indian Pines dataset.

Methods	DBDA	DBMA	FDSSC	SSRN	CEGCN	FDGCN	WFCG	CEGAT	SSFTT	Spectral Former	Proposed
1	92.53	82.75	93.01	82.28	48.96	78.1	96.23	97.92	95.6	0.49	**98.52 (0.0195)**
2	91.82	90.33	89.41	87.96	93.53	91.14	94	96.56	97.47	51.01	**98.73 (0.0053)**
3	94.95	92.94	87.54	91.19	90	94.86	93.24	97.06	96.12	54.86	**98.37 (0.0074)**
4	91.75	87.55	91.79	81.81	77.26	91.18	98.06	99.64	98.01	33.71	**97.91 (0.0189)**
5	98.13	93.47	98.93	96.11	90.4	94.59	94.21	96.15	97.2	81.4	**99.49 (0.0068)**
6	96.74	96.33	97.76	96.84	99.5	96.02	98.94	98.84	98.65	88.92	**99.82 (0.0006)**
7	66.55	53.13	81.77	82.18	50.8	66.61	96.53	**97.46**	91.2	0	93.5 (0.0491)
8	99.9	99.82	99.95	98.31	99.93	99.61	99.78	**99.97**	99.29	97.88	99.86 (0.0018)
9	72.18	60.75	64.07	76.89	26.45	73.18	92.77	**98.38**	66.67	0	85.56 (0.1295)
10	89.73	86.47	90.69	83.7	93.17	87.62	94.4	97.45	97.32	60.9	**97.62 (0.0155)**
11	96.17	92.14	94.39	87.43	97.58	93.27	97.69	98.39	98.06	78.17	**99.91 (0.0008)**
12	90.01	82.79	95.69	85.55	94.37	87.43	93.21	**98.85**	93.79	37.23	98.18 (0.0091)
13	94.98	94.99	97.74	95.67	**99.79**	93.37	98	99.24	99.78	82.94	99.24 (0.0073)
14	97.68	96.87	96.38	96.91	98.59	98.14	99.49	99.82	99.54	93.16	**99.95 (0.0011)**
15	91.25	84.4	92.97	87.14	86.99	94.02	98.7	**99.86**	96.85	21.99	98.35 (0.0141)
16	89.73	91.3	94.75	93.8	90.84	72.51	96.74	97.13	94.43	87.42	**99.03 (0.0141)**
OA	94.06	90.68	92.72	89.76	94.47	92.54	96.52	98.14	97.61	68.49	**99.1 (0.0019)**
AA	90.68	86.63	91.68	88.99	83.63	88.16	96.41	98.2	95	54.38	**98.75 (0.0099)**
KAPPA	93.24	89.38	91.71	88.3	93.68	91.48	96.03	97.88	97.27	63.71	**98.97 (0.0023)**

Note: The best results are highlighted in **bold**.

**Table 6:** Individual Class, OA, AA, and KPP of all methods on the Salinas dataset.

Methods	DBDA	DBMA	FDSSC	SSRN	CEGCN	FDGCN	WFCG	CEGAT	SSFTT	Spectral Former	Proposed
1	99.98	99.99	100	99.28	99.97	99.9	99.59	**100**	99.58	84.52	**100.0 (0.0000)**
2	99.97	99.97	100	99.62	100	99.6	99.96	**100**	100	96.94	**100.0 (0.0000)**
3	97.94	97.94	95.46	94.54	99.75	99.2	99.58	99.96	99.71	83.11	**100.0 (0.0000)**
4	93.52	91.92	96.56	93.97	99.19	90.68	98.82	99.63	97.99	97.44	**99.9 (0.0008)**
5	97.94	98.56	99.39	98.57	98.1	98.18	**98.63**	99.46	95.76	96.95	98.39 (0.0078)
6	99.94	98.47	99.89	99.85	99.85	99.01	99.87	99.12	99.97	97.93	**99.96 (0.0002)**
7	98.49	98.53	99.92	99.9	99.99	99.2	99.98	100	99.78	98.73	**100.0 (0.0000)**
8	93.52	93.66	91.85	82.73	97.28	98.41	97.41	98.72	97.28	81.88	**98.49 (0.0094)**
9	99.3	99.46	99.67	99.63	100	99.67	99.96	100	99.99	96.71	**100.0 (0.0000)**
10	98.52	95.97	97.29	95.69	**96.16**	97.83	97.76	98.45	97.76	79.87	97.68 (0.0103)
11	95.56	95.9	95.49	95.29	99.25	91.55	99.37	99.34	99.87	64.74	**99.54 (0.0061)**
12	99.43	98.99	98.69	98.27	**100**	94.58	99.99	100	99.92	93.97	99.97 (0.0006)
13	99.59	99.39	99.85	98.04	**99.87**	90.46	99.26	99.49	98.77	95.98	99.62 (0.0049)
14	96.36	95.99	96.73	96.65	98.76	89.22	97.13	**99.83**	98	86.45	98.03 (0.0175)
15	86.52	92.03	90.25	81.64	99.13	90.02	97.61	97.49	95.61	37.95	**99.25 (0.0063)**
16	99.88	99.15	99.66	99.63	99.43	99.26	99.55	**100**	99.35	87.43	**100.0 (0.0000)**
OA	94.88	96.43	96.16	92.42	98.89	96.63	98.76	99.18	98.32	82.78	**99.23 (0.0029)**
AA	97.28	97.25	97.54	95.83	99.17	96.05	99.03	99.47	98.71	86.29	**99.66 (0.0026)**
KAPPA	94.33	96.03	95.72	91.55	98.76	96.26	98.62	99.08	98.12	80.76	**99.15 (0.0033)**

Note: The best results are highlighted in **bold**.

**Table 7:** Individual Class, OA, AA, and KPP of all methods on the Pavia University dataset.

Methods	DBDA	DBMA	FDSSC	SSRN	CEGCN	FDGCN	WFCG	CEGAT	SSFTT	Spectral Former	Proposed
1	96.63	96.49	98.69	97.76	**99.07**	87.76	98.52	98.84	95.48	84.2	98.58 (0.0154)
2	99.37	98.93	99.23	98.34	99.96	99.02	99.95	100	99.76	93.44	**100.0 (0.0000)**
3	87.39	88.97	88.58	78.55	97.48	94.75	96.88	**98.92**	88.66	16.44	96.25 (0.0221)
4	96.44	96.71	99.47	99.67	95.07	79.42	95.35	**96.44**	90.85	80.79	96.29 (0.0145)
5	97.78	98.35	99.84	99.96	99.95	95.01	99.97	100	99.71	98.81	**100.0 (0.000)**
6	96.88	98.67	98.71	96.82	99.97	96.19	99.96	100	98.73	36.3	**100.0 (0.000)**
7	94.89	97.71	99.07	87.17	98.39	98.36	99.83	98.48	93.25	47.85	**99.92 (0.0012)**
8	86.73	88.34	88.43	86.26	97.11	79.8	98.58	96.81	96.22	84.29	**99.07 (0.0063)**
9	98.07	95.43	98.82	98.9	96.8	75.18	95.64	**99.57**	93.5	99.31	99.23 (0.0171)
OA	96.02	96.71	97.54	95.34	98.99	92.45	99.03	99.2	97.15	78.7	**99.33 (0.0018)**
AA	94.91	95.51	96.76	93.71	98.2	89.5	98.72	98.83	95.13	71.27	**98.94 (0.0032)**
KAPPA	94.74	95.63	96.74	93.82	98.66	89.98	98.72	98.9	96.22	71	**99.03 (0.0024)**

Note: The best results are highlighted in **bold**.

**Table 8:** Individual Class, OA, AA, and KPP of all methods on the WHU-Hi-LongKou dataset.

Methods	SVM	FNEA-OO	SVRFMC	CNNCRF	SSFTT	Spectral Former	Proposed
1	98.34	99.51	99.93	**99.99**	99.32	99.81	99.84 (0.0121)
2	93.62	99.90	**99.67**	99.08	79.30	99.40	**99.67 (0.0238)**
3	96.93	99.32	99.90	**100.0**	76.22	98.69	98.38 (0.0569)
4	87.46	97.72	96.56	97.79	97.83	99.69	**99.83 (0.0094)**
5	95.73	97.80	**99.43**	99.11	68.22	97.47	98.20 (0.0504)
6	99.23	99.67	99.97	99.80	97.72	99.51	**99.98 (0.0044)**
7	99.97	99.93	99.99	99.99	99.97	99.90	**100.0 (0.000)**
8	92.95	88.77	94.55	97.02	93.88	96.33	**97.98 (0.0882)**
9	92.42	94.74	86.66	91.07	86.60	93.15	**97.80 (0.0913)**
OA	94.96	98.59	98.37	98.91	96.67	99.41	**99.71 (0.0052)**
AA	95.18	97.48	97.41	98.21	88.78	98.22	**99.07 (0.0123)**
KAPPA	93.45	98.15	97.86	98.57	95.62	99.23	**99.62 (0.0069)**

Note: The best results are highlighted in **bold**.

**Table 9:** Model branch ablation study on the Salinas dataset.

Model Architecture	OA (%)	AA (%)	Kappa (%)	OA Difference (%)
Complete ESG-ViT	**99.23 ± 0.30**	**99.66 ± 0.26**	**99.15 ± 0.33**	Baseline
WP-ViT Only	96.84 ± 0.40	97.52 ± 0.22	96.48 ± 0.45	−2.39
GS-ViT Only	95.35 ± 0.38	91.96 ± 0.77	94.83 ± 0.42	−3.88

Note: The best results are highlighted in **bold**.

**Table 10:** LDA-SLIC scale parameter sensitivity study on the Salinas dataset.

Scale	OA (%)	AA (%)	Kappa (%)
50	99.19 ± 0.22	99.52 ± 0.21	99.05 ± 0.24
100	**99.23 ± 0.29**	**99.66 ± 0.26**	99.15 ± 0.33
150	99.20 ± 0.16	99.50 ± 0.16	**99.19 ± 0.18**
200	99.18 ± 0.14	99.46 ± 0.21	99.11 ± 0.16

Note: The best results are highlighted in **bold**.

**Table 11:** Quantitative comparison of computational cost, memory overhead, execution time, and classification accuracy on the Salinas dataset.

Model	Params (M)	FLOPs (G)	Training Time (s/epoch)	Test Inference Time (s)	Peak VRAM (GB)	OA (%)
CEGCN	0.167	66.274	0.0245	0.007	2.038	98.89
SSFTT	0.153	1164.069	0.0308	0.8579	0.029	98.32
SpectralFormer	0.366	6748.489	0.0802	2.8414	0.491	82.78
GS-ViT Only	0.815	138.539	0.0615	0.0199	1.751	95.35
WP-ViT Only	0.168	76.712	0.0634	0.0203	2.885	96.84
ESG-ViT	1.137	231.301	0.142	0.0412	4.974	99.23

## Data Availability

The data used in this study is publicly available in [34].
